# Editorial: Abiotic stress in plants: sustainability and productivity

**DOI:** 10.3389/fpls.2024.1386174

**Published:** 2024-03-20

**Authors:** Silvana de Paula Quintão Scalon, Cleberton Correia Santos, Maurizio Badiani, Luciane Almeri Tabaldi

**Affiliations:** ^1^ Faculty of Agricultural Sciences, Federal University of Grande Dourados, Dourados, Mato Grosso do Sul, Brazil; ^2^ Department of Agriculture, Mediterranea University of Reggio, Calabria, Italy; ^3^ Centre of Natural and Exact Sciences, Federal University of Santa Maria, Santa Maria, Rio Grande do Sul, Brazil

**Keywords:** antioxidant enzymes, photosynthetic metabolism, regulatory mechanisms, salinity, thermal stress, transcription factors, water stress

The Research Topic Abiotic Stress in Plants: Sustainability and Productivity was dedicated to study the effects of stressful conditions of abiotic origin, such as water and thermal stress, heavy metals and soil salinity, on crops and tree species, with focus on molecular and physio metabolic responses or adjustments. Innovative management and cultural treatments as a promising practice to induce tolerance and favor plant survival, as well as efficient recovery and productivity, were also considered in the thirteen articles published on this topic.

In their review, Yang et al. discuss recent advances in heat perception mechanisms in plants, with focus on second messengers. Furthermore, this review reported the regulatory mechanisms that involve specific transcription factors. Calcium ion, hydrogen peroxide, and nitric oxide have emerged as key players in heat perception. After discussing the roles of transcription, thermotolerance, and temperature targeting factors, such as plasma membrane-associated thermosensors, the authors paid attention to unsolved questions in the field of heat perception that require further investigation in the future.

The review by Qin et al. reports the potential of porous fiber materials (PFM) made from mineral rocks in conserving soil and water in areas where flooding and drought may occur. The effectiveness of PFM depends on their porosity and permeability, which, if high they can increase the capacity to retain, replace and infiltrate water into the soil during rainfalls, thereby reducing the risk of flooding, runoff and nutrients loss.

In two of the papers presented here, the issue of abiotic stress effects modulated by the plant’s developmental stage is examined. Zhang et al. observe that, at any stage, mild water deficit reduces the crop coefficient and evapotranspiration intensity in watermelon, and the water demand differs among the stages, increasing rapidly from the seedling stage to vines.

Similarly, Chen et al. describe that management of deficit irrigation is a strategy to conserve water in agriculture. In their study with sunflower, they also report that mild water deficit, both at the seedling stage and maturity, promoted increase in water use efficiency by leaves. The authors conclude that mild water deficit may be an effective irrigation strategy for sunflower production in the cold and arid environment of Northwest China.

In addition to the above focus on developmental stages, the research reported here shows that cultivars and genotypes of the same species respond differently to abiotic stresses as well as the resources used to mitigate adverse environmental conditions. In this context, Safarpour et al. report that, although considered as resistant plant species, barley plants exposed to water stress showed decreases in growth and grain production, as well as an enhanced antioxidants' response. The barley cultivar showing the highest proline content and catalase activity was the one with the highest biomass production and grain yield, which led to the conclusion that foliar urea applications can be effective to increase water stress tolerance, by improving the plant physiological performance.

Zhou et al. evaluated the effect of irrigation management strategies associated with nitrogen fertilization on eggplant, and verified that mild water deficit combined with medium nitrogen application rate (W1N2) is the right choice for growing conditions in arid environment, providing better water and nitrogen use efficiency and productivity.

The germplasm variability in stress responses was also studied by Li et al., who identified drought-resistant genotypes with potential value for breeding programs within a collection of 42 lettuce genotypes. Drought-resistance was found to be associated with little increases in stomata density, the production of superoxide and the content of malondialdehyde (MDA), but also with large increase in the activities of antioxidant enzymes, on the other hand.

In the same vein, Kumar et al. with the aim of identifying wheat genotypes tolerant to heat stress, verified that those tolerant ones maintained balanced phenological-physiobiochemical characteristics and high activities of their antioxidant enzymes. They also suggested that high photosynthesis and delayed senescence must be the best selection parameters for the heat tolerance in wheat.

The tolerance to a single stress or the combination of heat and drought stresses must be variable according to the cotton genotype, presenting different responses for enzymatic activity and non-enzymatic compounds (Zafar et al.). According to these authors, under combined stress, four genotypes exhibited superior performance in terms of agronomic traits and fiber quality, while others maintained gas exchange and relative water content, decreased their levels of H_2_O_2_ and MDA and increased levels of chlorophylls, carotenoids and activity of antioxidant enzymes.

Many papers reported here suggest that alternative cultural treatments can mitigate the stressful effects of the environment. In a study on the effect of partial replacement of chemical fertilizer (CF) by Trichoderma biofertilizer (TF) on wolfberry cultivation in saline lands,Yan et al. observed that replacement with 75% CF improves the N use efficiency and promotes higher photosynthetic rate, resulting in greater biomass and fruit production.

Seeking for the mitigation of water stress effects on ‘jatobazeiro’ seedlings (*Hymenaea courbaril* L.), Reis et al. confirmed the positive effect of intermediate shading of 30 and 50%, which accelerated the recovery of the photosynthetic rate after the resumption of irrigation. As a consequence, the cultivation under water deficit without shading (0%) should not be adopted for seedlings production in this species.

Considering that heavy metals pollution reduces the yield and quality of vegetables, Sun et al. demonstrated that the foliar spraying with zinc oxide nanoparticles (ZnO NPs) on tomato seedlings improved Cd tolerance, increased photosynthesis efficiency and antioxidant capacity, and reduced Cd accumulation in roots and leaves. Metabolomic analysis showed that exposure to ZnO NPs mitigates Cd toxicity, with higher effect on leaves than roots, with reduction of oxidative damage.

Once more in tomato seedlings, Turan et al. evaluated the potential of growth-promoting rhizobacteria (PGPR) to alleviate the stressful effect of drought, and verified that applying 4 L ha^-1^ of a biostimulant containing PGPR not only mitigated detrimental effects of water stress on hormonal balance and growth characteristics, but also restored plant growth and improved soil organic matter and the soil contents of total N, P, Ca, and Cu.

## Author contributions

SS: Writing – review & editing, Writing – original draft. CS: Writing – review & editing, Writing – original draft. MB: Writing – review & editing, Writing – original draft. LT: Writing – original draft.

